# Multi-decadal trends in contingent mixing of Atlantic mackerel (*Scomber scombrus*) in the Northwest Atlantic from otolith stable isotopes

**DOI:** 10.1038/s41598-021-86116-2

**Published:** 2021-03-23

**Authors:** Kohma Arai, Martin Castonguay, David H. Secor

**Affiliations:** 1 Chesapeake Biological Laboratory, University of Maryland Center for Environmental Science, Solomons, MD 20688 USA; 2 Fisheries and Oceans Canada, Institut Maurice-Lamontagne, Mont-Joli, QC G5H 3Z4 Canada

**Keywords:** Animal migration, Population dynamics, Stable isotope analysis, Ecology, Ecology, Environmental sciences

## Abstract

The Atlantic mackerel (*Scomber scombrus*) in the Northwest Atlantic is comprised of northern and southern components that have distinct spawning sites off Canada (northern contingent) and the US (southern contingent), and seasonally overlap in US fished regions. Thus, assessment and management of this population can be sensitive to levels of mixing between contingents, which remain unknown. Multi-decadal trends in contingent mixing levels within the US fisheries region were assessed, and the contingent composition across seasons, locations, ages, and size classes were characterized using archived otoliths and developing a classification baseline based on juvenile otolith carbon and oxygen stable isotopes (δ^13^C/δ^18^O values). Classification of age ≥ 2 adults demonstrated that northern contingent mixing was prevalent within the US continental shelf waters during the past 2 decades (2000–2019), providing an important seasonal subsidy to the US winter fishery despite substantial depletion in spawning stock biomass of the dominant northern contingent. While the majority of older fish were of the northern contingent during the early 2000s, the southern contingent contribution increased with age/size class during the recent period (2013–2019). Spatial mixing was most prevalent during February and March when the northern contingent occurred as far south as the Delmarva Peninsula, but were mostly absent from US waters in May. A positive relationship (albeit not significant; *r* = 0.60, *p* = 0.07) occurred between northern contingent mixing and US fisheries landings, which could imply that higher contingent mixing levels might be associated with greater landings for the US winter mackerel fishery. The yield of the Northwest Atlantic mackerel depends upon the status of the northern contingent, with the southern contingent possibly more prone to depletion. Spatially explicit stock assessment models are recommended to conserve both productivity and stability in this two-component population.

## Introduction

Complex spatial structure is a common attribute in fish populations^1–4^ that confers stability and resilience through asynchronous responses of population sub-components to the same environmental conditions (i.e., portfolio effect^5–7^). Yet fisheries stock assessment and management commonly relies on the unit stock concept—a traditional simplification of a group of fish within the same geographic area assumed to have homogenous internal dynamics (growth, mortality, reproduction) and limited exchange with other stocks^8^. Stock mixing occurs when stock separation is incomplete and distributions overlap across defined geographic boundaries over an extended period of time^9^. Applying the unit stock concept to a mixed-stock fishery can potentially lead to over exploitation of the subordinate spawning component as it receives a greater proportion of fishery removal relative to the more productive spawning component^8,10,11^. Additionally, ignoring spatial structure and stock mixing in assessment models can bias stock status indicators, resulting in flawed scientific advice^12–14^. Fishery stock assessment models and management units should therefore reflect the spatial structure of biological populations^15^.

Complex spatial structure has been reported for the Northwestern population of the Atlantic mackerel (*Scomber scombrus*)^16–18^, a migratory pelagic schooling species consumed around the globe, and a dominant forage fish species in the North Atlantic^19,20^. Two distinct stocks are recognized: one in the Northeast Atlantic off the coast of northern Europe, and another in the Northwest Atlantic centered off the US and Canada^21^. The Northeastern population has experienced an increase in abundance and geographical expansion of its distribution from the mid-2000s, currently supporting one of the largest fisheries in the world^22–24^. The Northwest Atlantic mackerel fisheries have fluctuated greatly over time, showing relatively high landings during the early 2000s, although the stock is currently in a depleted phase with spawning stock biomass and landings at historically low-levels^25,26^.

**Figure 1:**
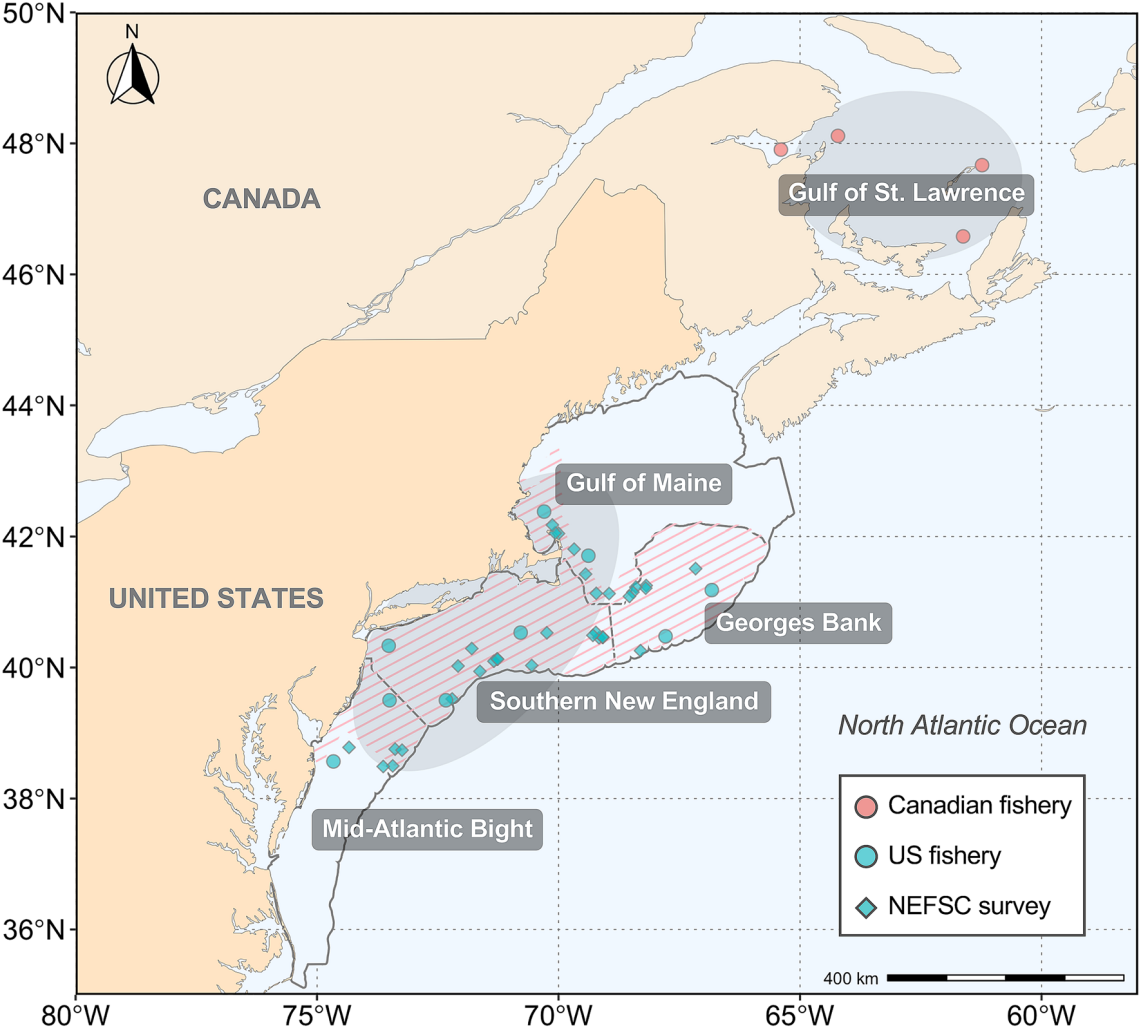
Map of the western North Atlantic Ocean illustrating sampling areas and spawning sites of the Northwest Atlantic mackerel. Sample locations are shown for the US winter fishery, the Canadian summer fishery, and the Northeast Fisheries Science Center (NEFSC) fishery-independent bottom trawl survey. Shaded ellipses in the Gulf of St. Lawrence and US continental shelf depict principal spawning sites for the northern and southern contingents. Hatched areas in the US continental shelf illustrate principal contingent mixing regions during winter. The map was created in R using the ggplot2 package version 3.3.2 URL: https://ggplot2.tidyverse.org^73^.

Defining the unit stock is crucial to fisheries management for the Northwest Atlantic mackerel, but a challenge given its highly dynamic migrations and broad spawning areas that transverse the border between the US and Canada, which separately manage jurisdictional fisheries. The population of the Northwest Atlantic mackerel has been traditionally broken into northern and southern “contingents” or sub-groups with different spawning sites, nursery areas, and migration behaviors^16,27^. The northern contingent centered in Canadian waters spawns primarily in the southern Gulf of St. Lawrence in June and July^28^ (Fig. 1), and the southern contingent spawns off southern New England in April and May^28,29^, although the spawning distribution and suitable habitat of the southern contingent has shifted northeastward over the past few decades^30–32^. The northern contingent migrates into the US shelf waters in late-fall and mixes with the southern contingent throughout late-fall and spring, where they are both exploited by the US winter fishery^14,16–18^ (Fig. 1). In late-spring, the northern contingent migrates back to spawn in the Gulf of St. Lawrence, and the two contingents are separated throughout the summer seasons^16,17^. As the biomass of the northern contingent is roughly an order of magnitude larger than that of the southern contingent^25,32^, the degree of contingent mixing likely influences US fisheries landings. However, contingent mixing levels and spatial structure of the Northwest Atlantic mackerel is highly uncertain and dynamic, and has only recently been quantified using otolith tracer approaches^18,33^.

Otolith oxygen and carbon stable isotopes (δ^18^O/δ^13^C values) have been widely used as a tool for addressing questions of population structure and migration of marine fishes^34–37^. Because the Northwest Atlantic mackerel are exposed to Northwest Atlantic shelf waters of differing temperature and water chemistry during their juvenile period^38,39^, this likely results in regional differences in the stable isotope composition in the otolith of the two contingents. Applying otolith oxygen stable isotope composition (δ^18^O values), Redding et al*.*^18^ discriminated between the two contingents by applying a year-class specific baseline approach, where a specific baseline was developed for each year-class to account for inter-annual variations in the stable isotope composition; then, unknown adult samples were aged and assigned to a year-class and matched with a corresponding year-class baseline. For the period of 2000–2003, the majority of age > 2 adults sampled from US shelf waters (in March) were classified as the northern contingent members, confirming the premise of seasonal mixing between the two contingents^18^. Moura et al*.*^33^ demonstrated a clear separation between the two contingents using a combination of bulk otolith chemistry and morphometrics, although sampling was both spatially and temporally restricted and from a single age-class.

In this study, contingent composition characteristics of the Northwest Atlantic mackerel from the recent periods (2013–2019) were compared with samples from the early 2000s^18^ to evaluate changes in contingent composition over multi-decadal timescales, and to assess how changes in spawning stock biomass and age structure influence contingent composition characteristics. Contingent mixing levels are likely to change over time due to contingent-specific production and migration patterns^9^. Further, the relationship between contingent mixing and US fisheries landings was examined to test whether US fisheries receive important seasonal subsidies from the dominant northern contingent. It was hypothesized that higher contingent mixing levels are associated with strong year-classes of the more dominant northern contingent, which will result in greater US fisheries landings, particularly during the winter period when contingent mixing is most prevalent^16^. The classification baseline of Redding et al*.*^18^ was updated by including otolith δ^13^C values as a second predictor, which reflects the carbon isotopic composition of dissolved inorganic carbon (DIC) and the diet source^40–42^, with the metabolic rate controlling the proportion of dietary carbon in the otolith in a temperature-dependent manner^43^. Further, instead of developing a specific baseline for each year-class^18^, a common classification baseline was developed by aggregating across year-class baselines^44^, and accounting for the inter-annual variations in the baseline stable isotope composition through a mixed effects modelling approach. The main goal of this study was to evaluate long-term trends in contingent mixing of the Northwest Atlantic mackerel within the principal US fisheries region, and to characterize contingent composition across seasons, locations, ages and size classes.

## Methods

### Sample collection

A total of 217 otolith samples were selected from the Northwestern population of the Atlantic mackerel caught in the US winter fishery from October to November and January to March during 2013–2019 in the Mid-Atlantic Bight (MAB), Southern New England (SNE), Georges Bank (GB), and Gulf of Maine (GOM; Fig. 1, Table S1). Fish collected in areas with high mackerel landings were selected for analysis. A total of 67 samples from the Northeast Fisheries Science Center (NEFSC) fishery-independent bottom trawl survey from March to May during 2013–2014, 2017, and 2019 were selected to augment age-1 juvenile baseline and age ≥ 2 adult samples for which US winter fishery samples were insufficient in sample size (Fig. 1). It is important to note that the sampling region of the NEFSC bottom trawl survey substantially overlaps with the US commercial mackerel fishery. A total of 99 otolith samples were selected from fish collected in the summer Canadian fishery from July to October during 2013–2016, and 2018 in the Gulf of St. Lawrence (Fig. 1). Samples were archived at the NEFSC Fisheries Biology Program in Woods Hole, Massachusetts, United States, and Fisheries and Oceans Canada (DFO), Maurice Lamontagne Institute, Mont-Joli, Québec, Canada. All samples had been aged by expert NEFSC and DFO scientists using whole otoliths mounted in clear resin viewed under reflected light. Ageing was generally conducted without prior information (e.g., fish length, weight) except for the date of capture. US commercial and survey samples collected in 2013–2019 achieved high ageing precision with the coefficient of variation (CV) ranging 0.0–1.5% and percent agreement ranging 95.7–100%. Month and location of capture, and length data were also available for almost all otolith samples. No live animals were used in this study and no specific permissions were needed for sampling activities as all otolith samples of Atlantic mackerel (not endangered nor protected) analyzed in this study represented archived material collected from commercial fisheries and government surveys.

### Otolith preparation and stable isotope analysis

Otolith oxygen and carbon stable isotope composition of age-1 juvenile samples were used to develop the baseline to classify age ≥ 2 unknown adult samples collected within the US shelf waters. Collection sites of age-1 juvenile samples were assumed to represent their natal habitats and exchange of individuals between the two contingents before the adult stage was presumed to be limited. This assumption is supported by evidence from size distribution analysis and extensive tagging programs that suggest localized migration patterns of age-0 juvenile mackerel^16,45^. Additionally, passive drift during the early life stage is predominately driven by wind forcing and occurs only for the short period before active swimming initiates at c. 20 days post hatch^28,46^. As age-1 juveniles of the northern contingent are rarely encountered by Canadian commercial fisheries, age-2 samples collected in the summer Canadian fishery in the Gulf of St. Lawrence were used to complement the 2016 year-class baseline for which age-1 samples of the northern contingent were insufficient in sample size. The use of age-2 fish as a baseline for the northern contingent is supported by evidence that immigration of the southern contingent into Canadian waters is not prevalent^16,17^. The otolith material within the first annulus representing the early life stage and residence within the natal nursery habitats was isolated and converted to a powder using a New Wave Research MicroMill (Fremont, California, USA), following the method of Redding et al.^18^. An automated series of 30 µm depth passes (10–13 passes) were made within the first annulus with a 500-µm carbide dental drill bit (Brasseler USA, Savannah, Georgia, USA) to extract the otolith material. The mass of powdered otolith material used for analysis ranged from 0.04 to 0.1 mg to achieve the best data quality.

δ^18^O and δ^13^C values of the otolith material were measured using an automated carbonate preparation device (KIEL-III; Thermo Fisher Scientific, Inc., Bremen, Germany) interfaced with a dual-inlet isotope ratio mass spectrometer (Finnigan MAT 252; Thermo Fisher Scientific, Inc., Bremen, Germany) at the University of Arizona’s Environmental Isotope Laboratory. Powered otolith material was reacted with dehydrated phosphoric acid at 70 °C under vacuum, and liberated CO_2_ was analyzed for stable isotope composition. All isotope values were reported in delta notation with respect to Vienna-Pee Dee Belemnite (V-PDB). Analytical precision of the mass spectrometer was calibrated based on repeated measurements of NBS-19 and NBS-18, and determined to be ± 0.10‰ for δ^18^O and ± 0.08‰ for δ^13^C (1 sigma).

### Data analysis

**Figure 2:**
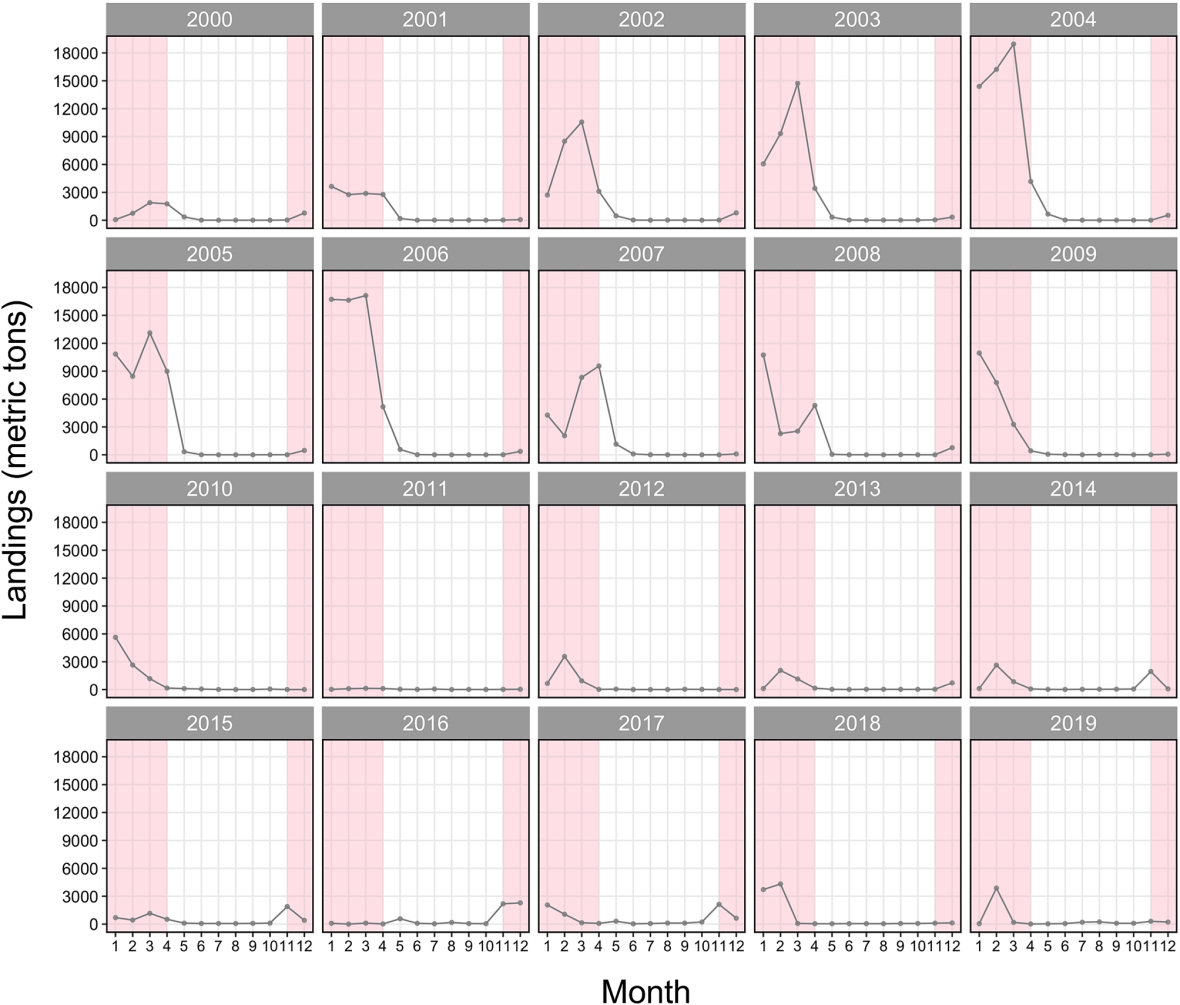
Monthly time series of US commercial mackerel landings from 2000 to 2019. Shaded backgrounds represent months with typically high landings (November–April). Data obtained from National Marine Fisheries Service, Northeast Fisheries Science Center (NMFS-NEFSC).

Classification baselines were developed separately for the older (1998–2000) and recent (2011–2016) year-classes due to a large shift in δ^18^O values (see results). Analyses for year-classes 1998–2000 and 2011 relied on data from Redding et al*.*^18^ but used a different classification procedure. Two-way multivariate analysis of variance (MANOVA) was used to test joint differences in the stable isotope composition (δ^18^O and δ^13^C values) of juvenile baseline samples across contingents (northern and southern), year-classes (2011–2016), and interaction. A separate two-way MANOVA was conducted to test joint differences in the otolith δ^18^O and δ^13^C values of US-collected juvenile samples across sub-regions (Gulf of Maine, Georges Bank, and Southern New England; Fig. 1), year-classes (2011–2016), and interaction. Significance of two-way MANOVAs was based on Pillai’s trace statistic. A binomial generalized linear mixed model (GLMM; *glmer* in the *lme4* package^47^) was fitted to the age-1 juvenile baseline data with a binomial distribution and logit link function. δ^18^O and δ^13^C values were used as fixed effects to estimate the probability of fish belonging to either contingent (northern or southern), and year-class was fitted as a random intercept in the model (i.e., the intercept is permitted to vary by year-class) to account for the interannual variation in the baseline stable isotope composition^48^. We also attempted to allow all predictors to have separate slopes for each year-class (i.e., random slopes), although increased model complexity resulted in (near) singular fits and almost no improvement in classification accuracy (< 1.5%). The parsimonious random intercept model was therefore accepted as the best model for contingent classification. Random oversampling (with replacement) was employed prior to model fitting to balance sample sizes for year-classes 2000 and 2011 baselines, in which the southern contingent made up > 60% of the baseline. For these year-classes, northern contingent data were randomly oversampled from the dataset within the specific year-class. Model diagnostics through residual inspections were performed with the *DHARMa* package^49^. Classification accuracy of the baseline model was assessed through tenfold cross-validation. In classifying baseline samples to each contingent, threshold classification probability levels of > 0.5 and > 0.7 were employed. Individuals that fell below the threshold probability level of > 0.7 were unassigned to either contingent. This baseline was then used to predict contingent membership (northern or southern) of age ≥ 2 unknown adult samples collected within the US shelf waters. For adult classification, a threshold classification probability level of > 0.7 was employed for probability of assignment to each contingent to account for the lack of strong separation between the two contingents in the baseline stable isotope composition^50^. Adult samples that fell below this threshold probability were unassigned to either contingent and were excluded from further analyses. Based on results of adult classification, contingent composition of Atlantic mackerel collected within US waters was characterized by year-class, age, size, and month and region of capture. For assessing contingent composition across regions, samples collected within each area during 2013–2019 were categorized into four broader regions: MAB, SNE, GB, and GOM (Fig. 1). Differences in frequencies of contingents across year-classes, age, size, and month of capture were assessed using Fisher’s exact tests with *p* values estimated through 2000 Monte Carlo simulations. The influence of contingent mixing on US commercial fisheries landings was examined by fitting a linear regression of log-transformed US commercial fisheries landings against estimated proportion of the northern contingent by year of capture through ordinary least squares minimization and assessed using Pearson’s correlation coefficient (*r*). Landings from November to April were used to represent months with high landings (Fig. 2) and when contingent mixing is expected to be most prevalent^16,17^. As peak landings have shifted to occur earlier in the season during the past 2 decades (Fig. 2), we selected the top three monthly landings during November to April to represent this historical winter fishery. We further assessed the relationship between contingent mixing levels and combined landings from July to October to test whether contingent mixing levels have no influence on landings during months when the northern contingent are expected to be absent from US waters. Statistical analyses were performed with R version 4.0.2^51^ with a significance alpha of 0.05.

## Results

### Baseline otolith stable isotope composition

**Figure 3:**
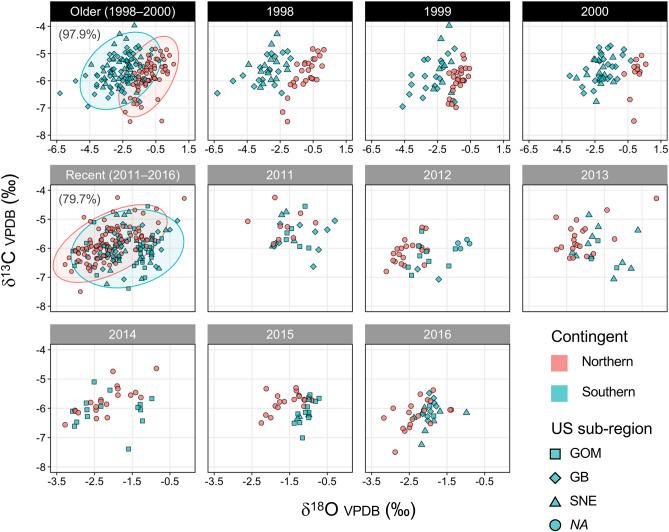
Otolith oxygen and carbon stable isotopes (δ^18^O/δ^13^C values) for Northwest Atlantic mackerel juvenile baseline samples collected in Canadian and US waters for older (black headers: 1998–2000) and recent (gray headers: 2011–2016) year-classes. The left plots in the first and second rows provide values for all year-classes combined with 95% confidence ellipses and cross-validated classification accuracy with threshold probability level of > 0.7 shown. Different symbols within US juvenile samples represent samples collected at different regions within US waters with *NA* indicating samples with no information on location of capture. All isotope values were reported in delta notation with respect to Vienna-Pee Dee Belemnite (V-PDB). Age-2 samples collected in the summer Canadian fishery in the Gulf of St. Lawrence were included in baselines for year-classes 1998, 1999, 2011, and 2016 to complement age-1 northern contingents that were insufficient in samples size. Note that the scales of the x-axis differ between older and recent samples. Data for year-classes 1998–2000 and 2011 are from Redding et al*.*^18^.

Among recent year-class samples, significant differences of baseline otolith δ^18^O and δ^13^C values were detected between contingents (Two-way MANOVA, *p*_contingent_ < 0.001; Fig. 3), and across year-classes (*p*_year-class_ < 0.001), but the interaction between contingent and year-class was not significant (*p*_contingent:year-class_ = 0.14). No significant sub-regional differences of baseline otolith δ^18^O and δ^13^C values were detected for US-collected juvenile samples (Two-way MANOVA, *p*_sub-region_ = 0.64, *p*_year-class_ < 0.001, *p*_sub-region:year-class_ = 0.53). Baseline otolith δ^18^O values showed a pronounced shift during the past 2 decades: the northern contingent exhibited higher values than the southern contingent for the older samples (1998–2000 year-classes), but showed lower values for the recent samples (2011–2016 year-classes). The two contingents exhibited similar δ^13^C values for the older samples, although the northern contingent exhibited higher values for some year-classes during the recent period (i.e., year-classes 2013 and 2015).

### Classification accuracy of the common classification baseline

Cross-validated mean classification accuracy of the mixed effects model was 79.7% for the recent (2011–2016), and 97.9% for the older (1998–2000) year-class baselines with a threshold probability level of > 0.7 (Table 1). For both baselines, including δ^13^C values as a second predictor and accounting for year-class effects as a random intercept in the model improved classification accuracy by 5.5% (recent) and 4.6% (older), and reduced the proportion of unassigned fish by 25.1% (recent) and 12.0% (older; Table1). For the older year-class baseline, classification accuracy of the common baseline approach (97.9%) was higher than that of the year-class specific approach which used a Random Forest procedure that relied only on δ^18^O for classification (range: 74.5–92.3%; Redding et al*.*^18^).

**Table 1:** Cross-validated mean classification accuracy and proportion of unassigned fish of the classification model that relied only on δ^18^O values and the mixed effects model which included δ^13^C values as a second predictor and accounted for year-class effects as a random intercept. Classification accuracy is shown for threshold classification probability level of > 0.7 (outside parenthesis) and > 0.5 (inside parenthesis). Classification accuracy of the year-class specific approach which used a Random Forest procedure that relied only on δ^18^O values is also shown^18^.

Model	Older (1998–2000 year-class)		Recent (2011–2016 year-class)	
	%classification accuracy	%unassigned	%classification accuracy	%unassigned
Contingent ~ δ^18^O	93.3 (88.0)	16.2	74.2 (62.9)	72.3
Contingent ~ δ^18^O + δ^13^C + (1|Year-class)	97.9 (96.9)	4.2	79.7 (69.8)	47.2
Random forest (Redding et al*.*^18^)	Range: 74.5–92.3%	–	–	–

### Contingent mixing on the US continental shelf and contingent composition characteristics

**Figure 4:**
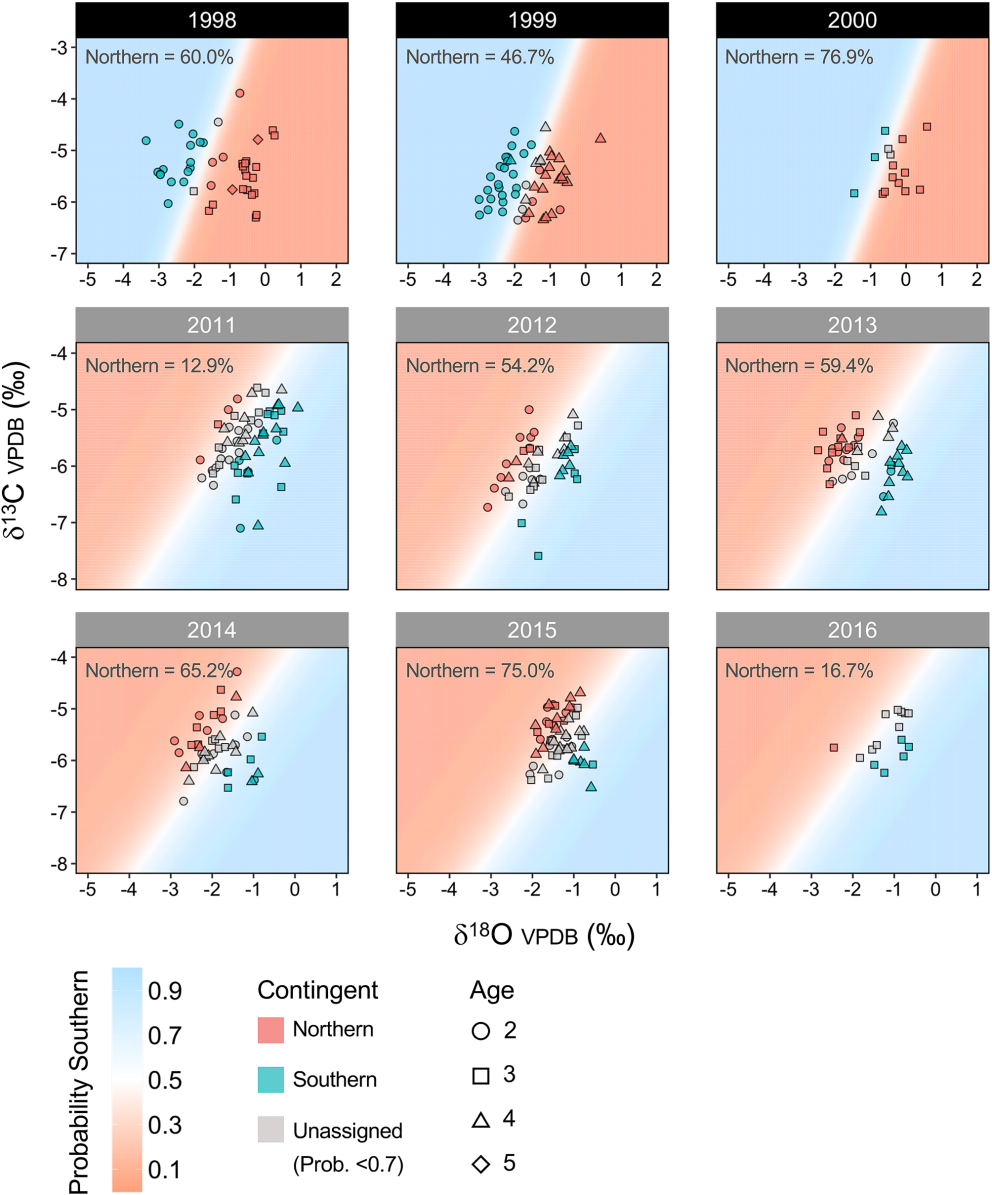
Otolith oxygen and carbon stable isotopes (δ^18^O/δ^13^C values) for age ≥ 2 unknown adult Northwest Atlantic mackerel samples collected in the US shelf waters for 9 year-classes (1998–2000 and 2011–2016) classified based on the binomial generalized linear mixed model. Background colors indicate the probability of each individual assigned to either contingent (0.7 threshold). Different symbols represent different age-classes within each year-class. The proportion of the northern contingent of assigned fish (probability > 0.7) for each year-class is shown in the top left corner of each panel. All isotope values were reported in delta notation with respect to Vienna-Pee Dee Belemnite (V-PDB). Note that classification baselines were developed separately for the older and recent year-classes and the scales of the x- and y-axes differ between those panels.

A threshold classification probability level of > 0.7 resulted in an exclusion of 12 and 123 (10.9% and 46.1% of total samples) age ≥ 2 adult samples from the older and recent year-classes. Additionally, a probability level of > 0.5 was tested (resulting in no excluded individuals), which resulted in no to small changes (< 10%) in contingent membership across most categories of interest (Table S2). While moderate changes (> 15%) in contingent memberships occurred in few categories with small sample size, contingent composition characteristics were generally consistent between two threshold probability levels. Classification of age ≥ 2 adults based on otolith carbon and oxygen stable isotopes indicated that mixing between the northern and southern contingents was prevalent during the past 2 decades in US shelf waters (Fig. 4). Contingent membership frequencies of assigned samples (probability > 0.7) were significantly different across recent year-classes (Fisher’s exact test, *p* < 0.001). Among assigned samples, the northern contingent was dominant for the 2015 year-class (75% northern), but the southern contingent dominated year-classes 2011 (87% southern), and 2016 (83% southern).

**Figure 5:**
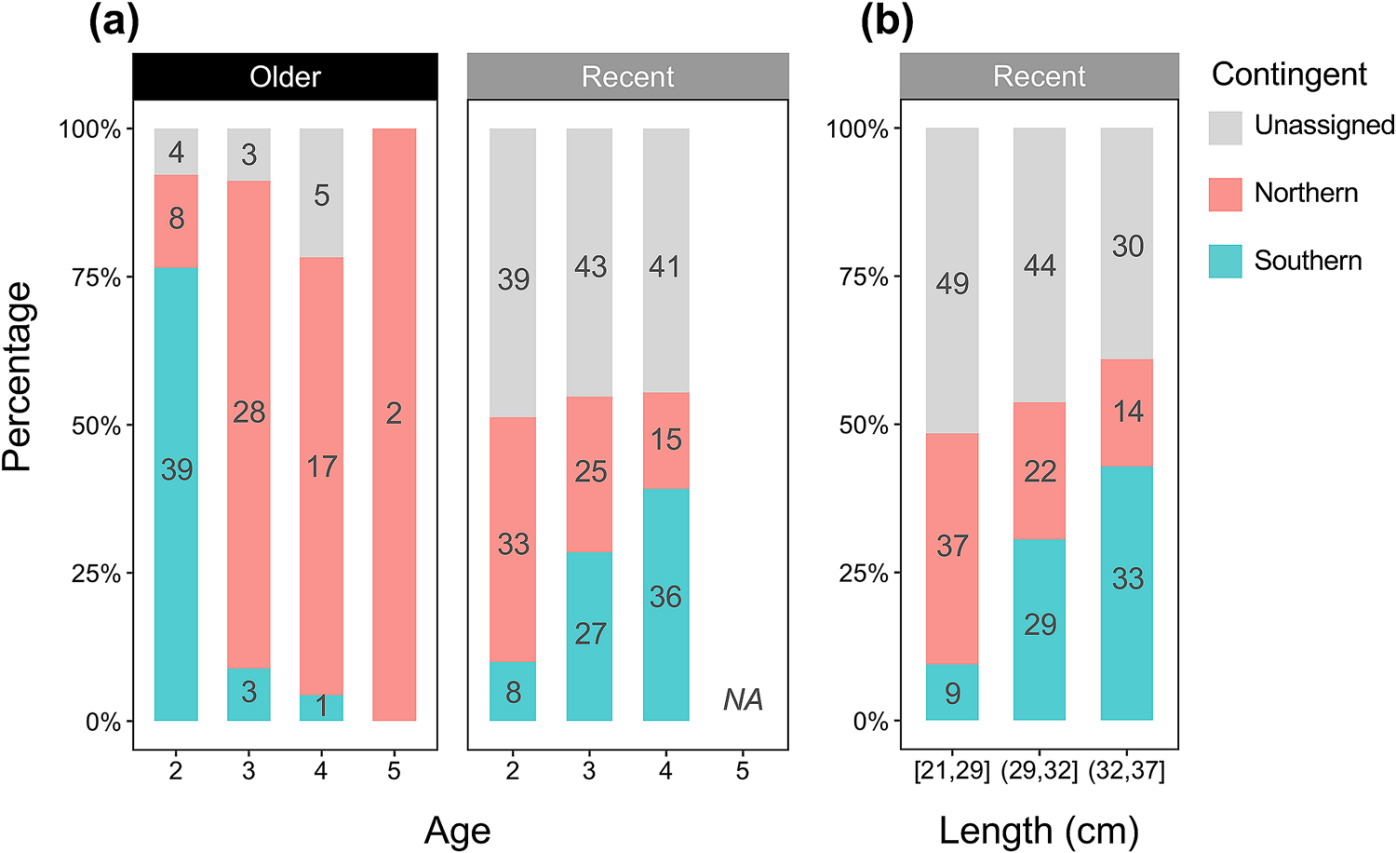
Contingent composition of Northwest Atlantic mackerel age ≥ 2 adult samples across (**a**) age class for older (1998–2000) and recent (2011–2016) year-classes, and (**b**) size class (cm) for the recent year-class samples. Numbers in each bar plot indicate the sample size of northern and southern contingents, and unassigned fish (probability < 0.7) for each age and size class.

Contingent composition estimated by age-class for recent year-classes indicated that among individuals that met the probability threshold level of > 0.7, age-2 fish were composed largely of the northern contingent, but ages 3 and 4 were more likely to be of the southern contingent (Fisher’s exact test, *p* < 0.001; Fig. 5a). This pattern is in stark contrast to that of the year-classes drawn for the earlier period where age-2 fish were dominated by the southern contingent, and the majority of ages 3 and 4 were of the northern contingent (Fisher’s exact test, *p* < 0.001; Fig. 5a). Contingent frequencies were significantly different across size for the recent year-classes (Fisher’s exact test, *p* < 0.001), in which the proportion of the southern contingent increased with fish length (Fig. 5b).

**Figure 6:**
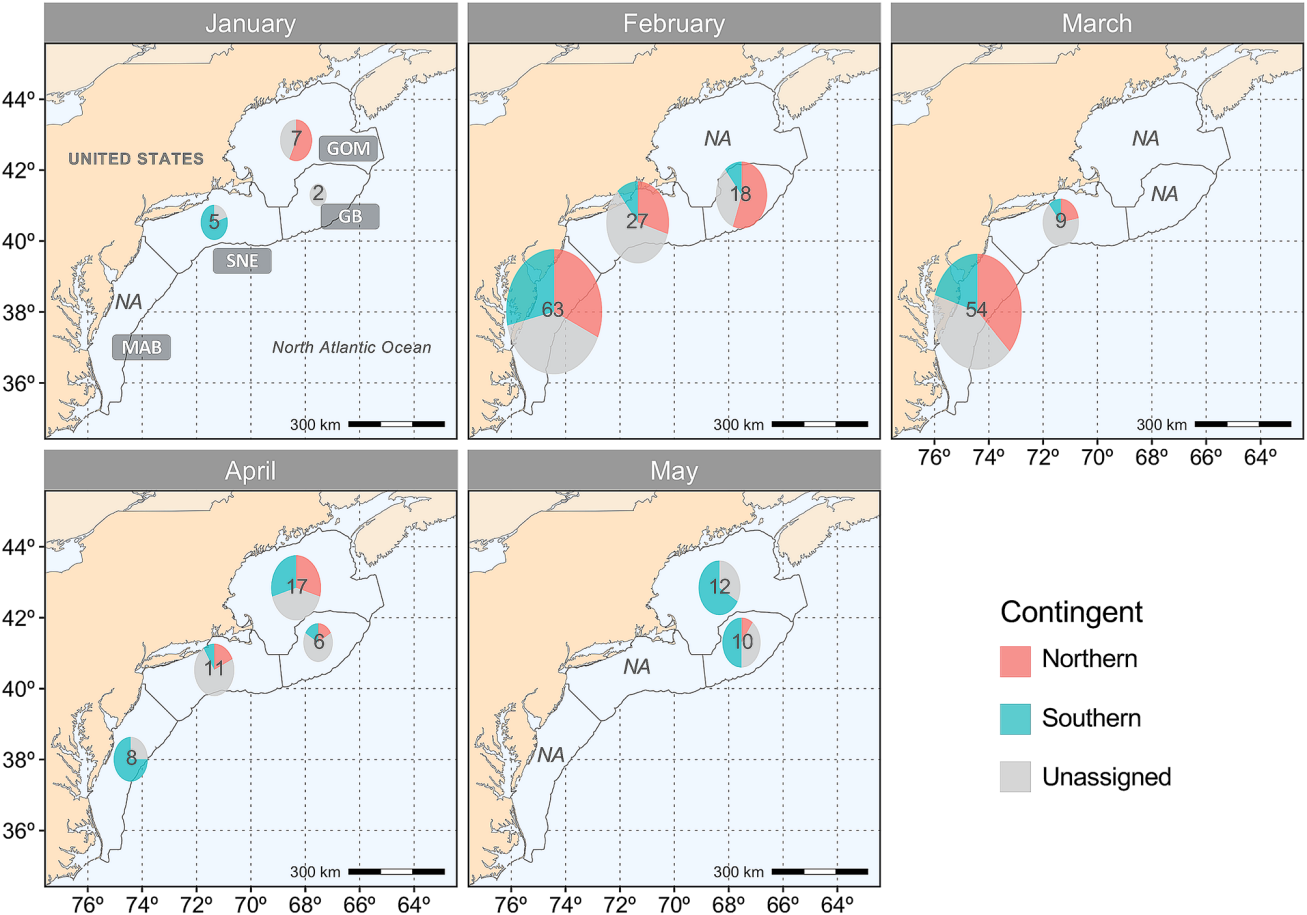
Contingent composition of Northwest Atlantic mackerel age ≥ 2 adult samples by region at capture through January and May collected in 2013–2019. The four regions (MAB, SNE, GB, GOM) are shown in outlined boxes. Each pie chart indicates the number of samples collected within each region, and is proportional to the sample size. The map was created in R using the ggplot2 package version 3.3.2 URL: https://ggplot2.tidyverse.org^73^.

Estimated contingent composition by region at capture for recent year-classes indicated that during the period of January to May, the northern contingent co-occurred with the southern contingent off the Delmarva Peninsula (Delaware, USA) in the south, to the Gulf of Maine in the north (Fig. 6). Significant differences in contingent frequencies among assigned samples (probability > 0.7), were found across months at all regions (Fisher’s exact test, *p*_*MAB*_ < 0.05; *p*_*GB*_ < 0.05; *p*_*GOM*_ < 0.01), except for the SNE (Fisher’s exact test, *p*_*SNE*_ = 0.07). In January, samples from SNE (*n*_south_ = 4, *n*_north_ = 0) were dominated by the southern contingent, with all samples from the GOM (*n*_south_ = 0, *n*_north_ = 4) represented by the northern contingent. The majority of samples in February in the MAB (*n*_south_ = 18, *n*_north_ = 20), SNE (*n*_south_ = 3, *n*_north_ = 8), and GB (*n*_south_ = 2, *n*_north_ = 10) were of the northern contingent, with a greater proportion of the northern contingent occurring in the SNE than in the previous month. The northern contingent further dominated samples from the MAB (*n*_south_ = 11, *n*_north_ = 20) and SNE (*n*_south_ = 1, *n*_north_ = 2) in March. The southern contingent dominated sample composition in the MAB (*n*_south_ = 6, *n*_north_ = 0) during April, although samples caught in SNE (*n*_south_ = 1, *n*_north_ = 2), GB (*n*_south_ = 1, *n*_north_ = 1) and GOM (*n*_south_ = 5, *n*_north_ = 5) were composed of nearly equal proportions of both contingents. In May, nearly all samples collected in GB (*n*_south_ = 5, *n*_north_ = 1) and GOM (*n*_south_ = 8, *n*_north_ = 0) were exclusively from the southern contingent.

**Figure 7:**
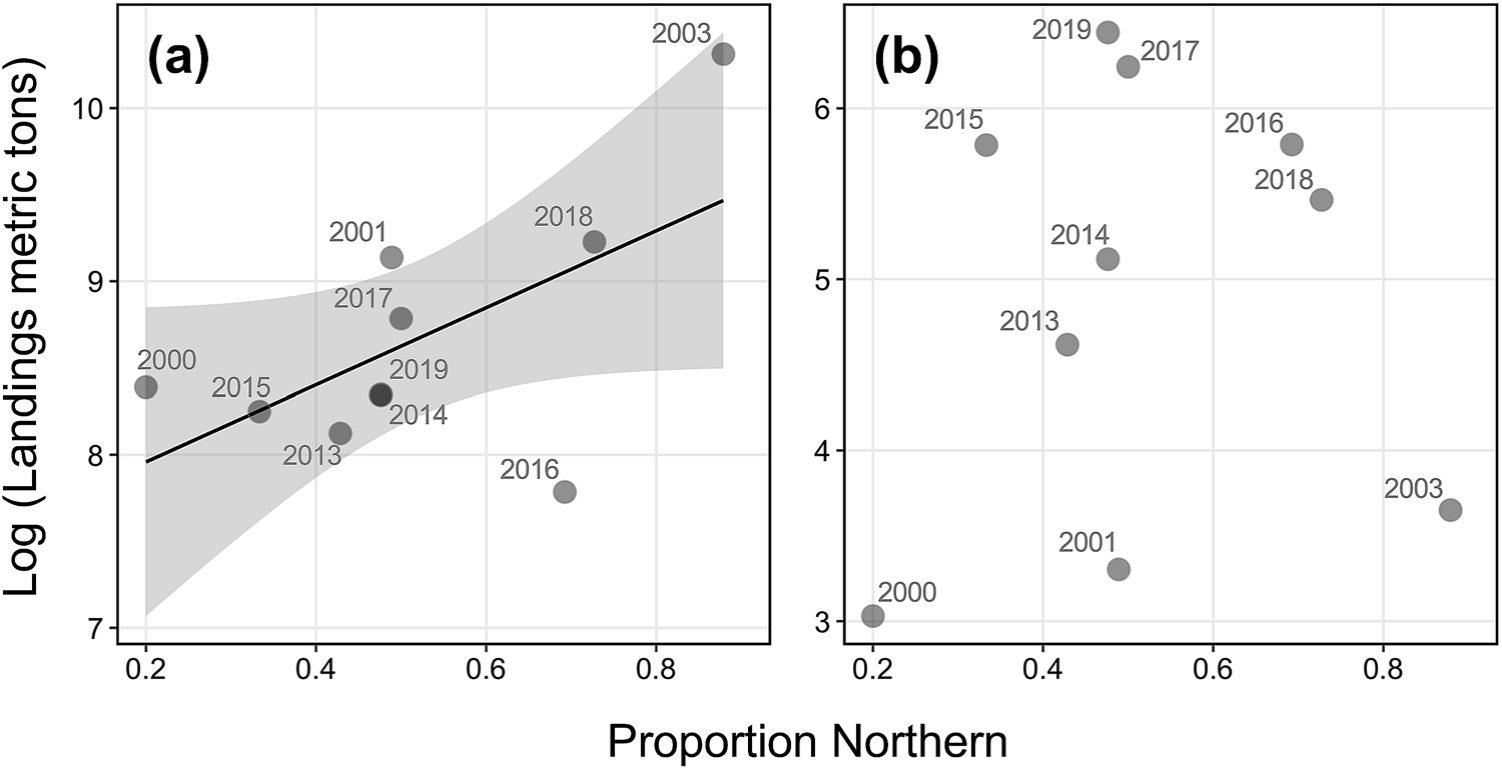
Relationship between the estimated annual proportion of northern contingents of assigned fish (probability > 0.7) occurring within the US shelf waters and (**a**) combined top three monthly US commercial landings during November to April and (**b**) combined landings from July to October. Data points are labeled by year of capture. The black line indicates the linear regression line fitted to the data and the shaded area indicates 95% confidence intervals. Note that the scale of the y-axis differs among panels.

A positive, although not statistically significant relationship (Pearson’s *r* = 0.60, *p* = 0.07, *n* = 10), was observed between the estimated proportion of northern contingent in the US shelf waters (age ≥ 2) and landings during peak winter months, with larger residuals occurring for high stock mixing levels (Fig. 7a). No relationship was apparent (Pearson’s *r* = 0.10, *p* = 0.78, *n* = 10) between northern contingent contribution and landings from July to October (Fig. 7b).

## Discussion

Evaluation of contingent mixing of Northwest Atlantic mackerel revealed decadal changes in the relative availability of the northern contingent to US fisheries. Despite substantial decrease in spawning stock biomass of the dominant northern contingent^26^ (Fig. S1a), contingent mixing persisted during the past 2 decades (2000–2019), providing an important seasonal subsidy to the US commercial fishery, particularly during the winter months when peak landings occur. Still, the relative contribution of the northern contingent has diminished in some year-classes during the most recent decade.

Spatial and temporal trends in contingent composition among samples collected in 2013–2019 corresponded well with traditional views on seasonal migration patterns and population structure of the Northwest Atlantic mackerel^16,17,25,52^. Some degree of spatial segregation occurred between the two contingents in January, consistent with the onset of the southerly (fall) migration and overwintering by the northern contingent in US shelf waters (Fig. 6). Spatial mixing was strongest during February and March when the northern contingent occurred as far south as the Delmarva Peninsula, which matches results from tag-recapture studies^17^. These months correspond well with periods when peak landings occur in the US fishery (Fig. 2), most likely because the northern contingent are subjected to exploitation in those regions. The near absence of the northern contingent from the MAB in April, and from the GB and GOM in May aligns with the departure of the northern contingent from the US continental shelf waters to make their northerly (spring) migration towards the spawning sites in the Gulf of St. Lawrence. Interestingly, during part of the spawning season, April, the northern contingent co-occurred with the southern contingent, which could provide support for straying between the two contingents should the northern contingent be spawning within US waters^53^.

Otolith stable isotope composition analysis presented here and by Redding et al*.*^18^ indicate prevalent contingent mixing within the US continental shelf during the past 2 decades (2000–2019). Contingent mixing levels of the Northwest Atlantic mackerel within the US continental shelf could be influenced by changes in migration behaviors and/or relative abundance differences between the two contingents^9^. Distribution shifts and range expansion by Atlantic mackerel has been associated with changes in oceanographic conditions^30,54^ and stock size^22,23^. Yet the apparent winter distribution of the northern contingent was consistent with historical distributions^17^ (Fig. 6), and contingent mixing persisted during the past 2 decades despite substantial depletion in spawning stock biomass (SSB) of the dominant northern contingent^26^ (Fig. S1a). Still, the apparent disappearance of older/larger members of northern contingents during the recent decade (2013–2019; Fig. 5) likely reflects depleted stock status and severe age truncation experienced by the northern contingent^26^.

Contingent mixing levels were highly variable among year-classes: the northern contingent dominated sample composition of the 2015 year-class, but the majority of samples for year-classes 2011, and 2016 were of the southern contingent (Fig. 4). This variation could be partially explained by the recruitment strength of the northern contingent, in which high mixing levels in the 2015 year-class samples was attributed to the moderately strong 2015 year-class, and low mixing levels of the 2016 year-class reflecting historically low recruitment levels^26^ (Fig. S1b). The strongest year-class in the past 2 decades occurred in 1999^55^, although recruitment strength was not reflected in the contingent composition for that year-class (Fig. 4: 46.7% northern). This inconsistency could be due to relatively strong egg and larval productions of the southern contingent during the early 2000s^32,56^. While contingent mixing levels are likely strongly influenced by relative recruitment strengths of both contingents, recruitment estimates for the southern contingent remains highly uncertain given that the US assesses both contingents as a unit stock, whereas Canada assesses the northern contingent in isolation. Still, recruitment strength of the northern contingent should have a larger effect on the observed stock mixing dynamics as the northern contingent is more productive than the southern contingent^25^. It is also important to note that contingent mixing levels within each year-class could be influenced by the unbalanced sample size of age-classes for some year-classes (e.g., 2000, 2016), given the observed ontogenetic trends in contingent mixing levels (Fig. 5a).

The hypothesis of whether contingent mixing levels are associated with US landings during the winter period was tested. A positive correlation (albeit not significant) occurred between the northern contingent contribution and landings during peak winter months when contingent mixing is expected to be most prevalent (Fig. 7a). Further, in line with our expectations, contingent mixing levels had negligible influence on landings during periods when northern contingents should be absent from the US waters (July–October). While small sample size precludes strong inferences, these results could imply that higher contingent mixing levels might be associated with greater landings for the US winter mackerel fishery, such as was observed for the strong 2015 northern contingent year-class (year of capture: 2017 and 2018). It is also worth highlighting that the US winter fishery has shifted from a multi-month fishery through January to April (2000–2010), to one which depends on a single-month pulse during November to February (2011–2019; Fig. 2). This transition towards a single-month pulse fishery during the recent decade (2011–2019) could be indicative of the diminished stock status of the northern contingent, and could be one reason for the lack of a strong relationship between northern contingent contribution and US winter landings. Additionally, fluctuations in mackerel landings also reflect factors that are independent of mackerel availability including regulations related to the incidental catches of river herring/shad (*Alosa* spp.), market demands, fuel costs, and fleet and processing capacity.

Otolith stable isotope composition (δ^18^O/δ^13^C values) baselines for the recent year-class samples (2011–2016) exhibited significant differences between the two contingents (Fig. 3), which follows previous studies that have successfully discriminated between the two contingents through otolith tracer approaches^18,33^. Significant temporal variability occurred in the baseline stable isotope composition, which could be due to regional shifts in temperature and water chemistry. Notably, baseline otolith δ^18^O values exhibited a pronounced shift during the past 2 decades (2000–2019; Fig. 3). Interdecadal shifts in the baseline otolith stable isotope composition has been reported for Atlantic bluefin tuna, attributed to the decrease in δ^13^C values of atmospheric CO_2_ and increase in seawater δ^18^O values in the Atlantic Ocean over the past several decades^57,58^. Because otolith aragonite forms close to isotopic equilibrium with the oxygen isotope composition of ambient water^40,59,60^, the decadal shift in otolith δ^18^O values could be associated with changes in large-scale oceanographic drivers including the Atlantic meridional overturning circulation (AMOC^61^), which alters seawater composition within the US continental shelf^62^. Accelerated warming trends within the US continental shelf waters from the late 2000s^63,64^ could also influence natal otolith δ^18^O values through temperature-dependent fractionation^40,59^. Failing to incorporate such temporal variability in the baseline stable isotope composition could confound the performance of stock classification.

Temporal variability in otolith chemistry often requires (1) matching of unknown adults to a corresponding year-class baseline^18,65,66^; or (2) aggregating across multiple year-classes to fully represent temporal variation within the baseline^44^. We adopted a hybrid approach through application of a mixed effects model, in which the intercept was permitted to vary by year-class^48^. Classification accuracy was further improved by incorporating δ^13^C values as a second predictor, which could be indicative of regional differences in the carbon stable isotope composition of the food source and/or dissolved inorganic carbon (DIC) in seawater^40–42^, as well as differences in contingent-specific metabolic rates during their juvenile growth period^43,67,68^.

Results on stock composition can vary based on the classification threshold probability of choice. A threshold probability level of > 0.7 was employed for individual assignment to account for the incomplete separation between the baseline stable isotope composition of the two contingents, although caution must be exercised when estimating stock composition through individual assignment as it ignores uncertainty associated with each individual assignment. Future studies should consider employing mixture analysis which estimates stock composition jointly on all individuals rather than summing independent assignments^69^. It is also important to note that biases could arise from incomplete spatiotemporal sampling, although the majority of samples in this study were collected in areas with high mackerel landings and during months when contingent mixing was expected to be most prevalent^16,17^. Analyzing samples from both the spring and fall NEFSC bottom trawl survey should be valuable next steps to fully uncover the seasonal migration patterns of the Northwest Atlantic mackerel, and to understand any potential sampling bias. Further, given that contingent mixing is highly dynamic and is likely to change over longer time scales^70^, future efforts should focus on analyzing samples during periods of exceptionally high landings (i.e., 1970s) when southern contingent contribution to the overall population was expected to be high^32^. Additionally, while contingent mixing is more likely to occur within the US fishery^16^, it is important to assess the contribution of the southern contingent to the Canadian fishery and whether this can change over time.

Harvesting a mixed-stock comprised of multiple population sub-components of unique demographics under a unit stock concept can potentially lead to overexploiting the less productive component as it receives a greater proportion of fishery removal than the productive component^8,10,11^. It is important to guard against loss of such minority components as they can contribute to population stability and resilience through the “portfolio effect”^7,71^. Information on contingent composition could further help develop spatially explicit stock assessment models for the Northwest Atlantic mackerel population to provide advice designed to conserve both contingents^8,14,72^. Observed contingent mixing among the Northwest Atlantic mackerel during the past 2 decades (2000–2019) provides support for incorporating spatial structure in stock assessment and management which could potentially conserve both productivity and stability of this depleted two-component population.

## Supplementary Information

Supplementary Figure.

Supplementary Information.
