# Supplementary figures and images for: Multi-decadal trends in contingent mixing of Atlantic mackerel (Scomber scombrus) in the Northwest Atlantic from otolith stable isotopes
Source: Sci Rep. 2021 Mar 23;11:6667. doi: 10.1038/s41598-021-86116-2 (PMC7988008; doi:10.1038/s41598-021-86116-2)

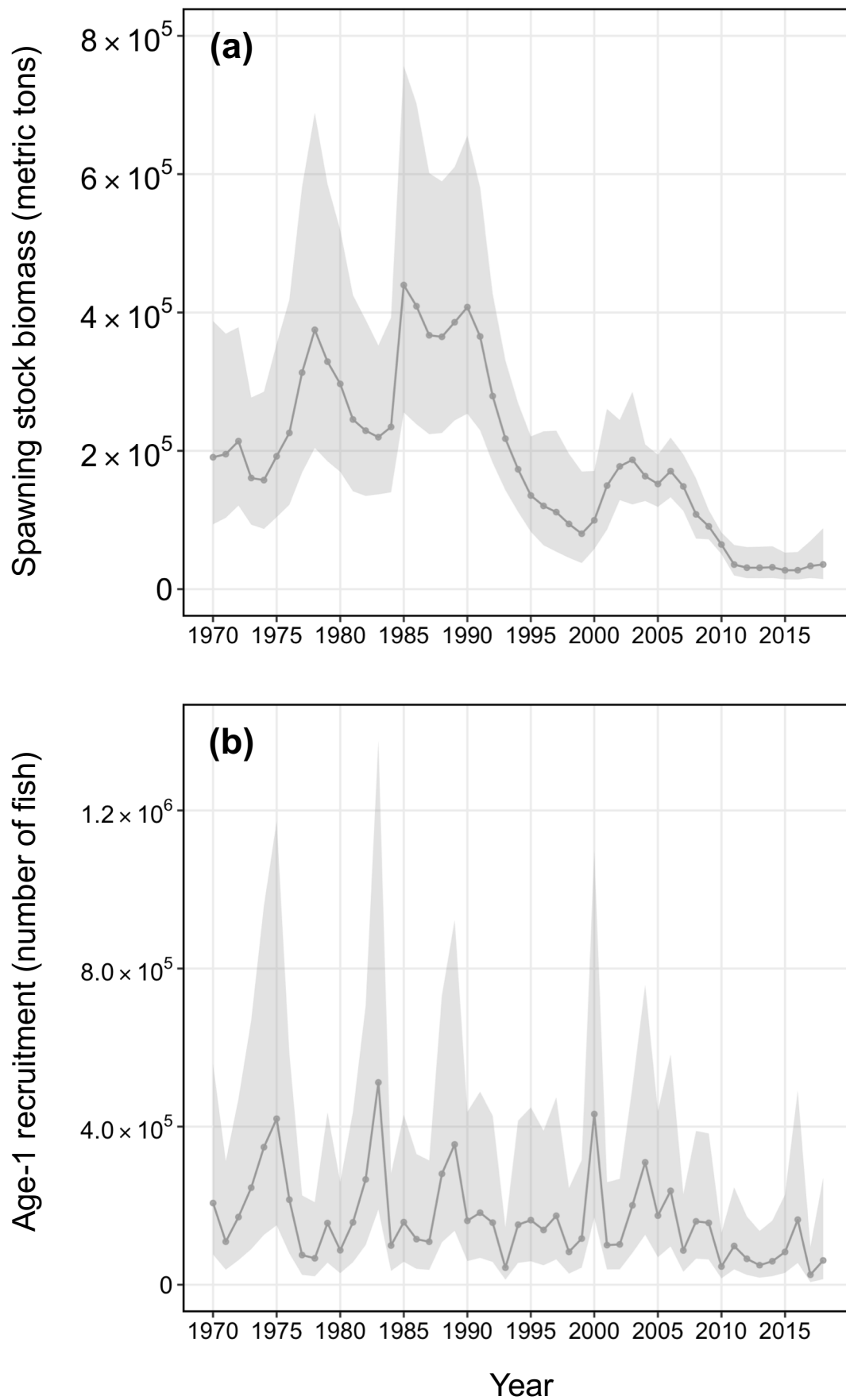

**Arai et al. Supplementary Figure 1**

Supplement: Supplementary file 1 — Supplementary Figure. [file 41598_2021_86116_MOESM1_ESM.pdf]
