# Supplementary material for: Multi-decadal trends in contingent mixing of Atlantic mackerel (Scomber scombrus) in the Northwest Atlantic from otolith stable isotopes
Source: Sci Rep. 2021 Mar 23;11:6667. doi: 10.1038/s41598-021-86116-2 (PMC7988008; doi:10.1038/s41598-021-86116-2)
Supplement: Supplementary file 2 — Supplementary Information. [file 41598_2021_86116_MOESM2_ESM.docx]

# **Supplementary material**

Multi-decadal trends in contingent mixing of Atlantic mackerel (*Scomber scombrus*) in the Northwest Atlantic from otolith stable isotopes

Kohma Arai^1*^, Martin Castonguay^2^, David H. Secor^1^

^1^Chesapeake Biological Laboratory, University of Maryland Center for Environmental Science, Solomons, MD 20688, USA

^2^Fisheries and Oceans Canada, Institut Maurice-Lamontagne, Mont-Joli, QC, G5H 3Z4 Canada

^*^Corresponding author

Kohma Arai

Email: [karai@umces.edu](mailto:karai@umces.edu)

E-mail addresses:

Martin Castonguay: [Martin.Castonguay@dfo-mpo.gc.ca](mailto:Martin.Castonguay@dfo-mpo.gc.ca)

David H. Secor: [secor@umces.edu](mailto:secor@umces.edu)

**Supplementary Table 1** Northwest Atlantic mackerel otolith sample size by year-class, sampling period, collection site, and age-class. Data for year-classes 1998–2000 and 2011 are from Redding *et al*.^18^.

| **Year-class** | **Years collected** | **Collection site** | **Age** | | |  | | |
| --- | --- | --- | --- | --- | --- | --- | --- | --- |
|  |  |  | **1** | **2** | **3** | | **4** | **5** |
| 1998 (N = 102) | 1999–2001, 2003 | Canada | 15 | 13 |  | |  |  |
|  |  | Gulf of Maine |  |  |  | |  |  |
|  |  | Georges Bank | 18 | 10 | 7 | |  |  |
|  |  | Southern New England | 14 | 11 | 7 | |  | 2 |
|  |  | Mid-Atlantic Bight |  |  | 5 | |  |  |
|  |  | NA |  |  |  | |  |  |
| 1999 (N = 107) | 2000, 2001, 2003 | Canada | 12 | 15 |  | |  |  |
|  |  | Gulf of Maine |  |  |  | |  |  |
|  |  | Georges Bank | 19 | 14 |  | | 4 |  |
|  |  | Southern New England | 8 | 16 |  | | 16 |  |
|  |  | Mid-Atlantic Bight |  |  |  | | 3 |  |
|  |  | NA |  |  |  | |  |  |
| 2000 (N = 66) | 2001, 2003 | Canada | 12 |  |  | |  |  |
|  |  | Gulf of Maine |  |  |  | |  |  |
|  |  | Georges Bank | 24 |  | 2 | |  |  |
|  |  | Southern New England | 15 |  | 7 | |  |  |
|  |  | Mid-Atlantic Bight |  |  | 6 | |  |  |
|  |  | NA |  |  |  | |  |  |
| 2011 (N = 89) | 2012–2015 | Canada |  | 9 |  | |  |  |
|  |  | Gulf of Maine | 6 | 7 |  | | 16 |  |
|  |  | Georges Bank | 10 | 4 | 10 | |  |  |
|  |  | Southern New England | 4 |  | 2 | | 4 |  |
|  |  | Mid-Atlantic Bight |  | 9 | 8 | |  |  |
|  |  | NA |  |  |  | |  |  |
| 2012 (N = 80) | 2013–2016 | Canada | 20 |  |  | |  |  |
|  |  | Gulf of Maine | 9 |  |  | | 7 |  |
|  |  | Georges Bank | 2 | 15 |  | | 3 |  |
|  |  | Southern New England |  |  |  | | 2 |  |
|  |  | Mid-Atlantic Bight |  |  | 15 | | 3 |  |
|  |  | NA | 4 |  |  | |  |  |
| 2013 (N = 79) | 2014–2017 | Canada | 20 |  |  | |  |  |
|  |  | Gulf of Maine | 3 |  |  | |  |  |
|  |  | Georges Bank |  |  |  | |  |  |
|  |  | Southern New England | 11 |  |  | |  |  |
|  |  | Mid-Atlantic Bight |  | 15 | 15 | | 15 |  |
|  |  | NA |  |  |  | |  |  |
| 2014 (N = 80) | 2015–2018 | Canada | 20 |  |  | |  |  |
|  |  | Gulf of Maine | 15 |  |  | |  |  |
|  |  | Georges Bank |  |  |  | |  |  |
|  |  | Southern New England |  |  |  | | 12 |  |
|  |  | Mid-Atlantic Bight |  | 15 | 15 | | 3 |  |
|  |  | NA |  |  |  | |  |  |
| 2015 (N = 92) | 2015–2019 | Canada | 20 |  |  | |  |  |
|  |  | Gulf of Maine | 11 | 7 |  | | 5 |  |
|  |  | Georges Bank |  |  |  | | 2 |  |
|  |  | Southern New England | 4 |  | 15 | | 13 |  |
|  |  | Mid-Atlantic Bight |  | 8 |  | | 3 |  |
|  |  | NA |  |  |  | | 4 |  |
| 2016 (N = 52) | 2017–2019 | Canada |  | 19 |  | |  |  |
|  |  | Gulf of Maine | 2 |  | 1 | |  |  |
|  |  | Georges Bank | 8 |  | 2 | |  |  |
|  |  | Southern New England | 8 |  | 4 | |  |  |
|  |  | Mid-Atlantic Bight |  |  | 1 | |  |  |
|  |  | NA |  |  | 7 | |  |  |

**Supplementary Table 2** Summary contingent composition of Northwest Atlantic mackerel age ≥2 adult samples by year-class, age-class, size-class, and region of capture through January to May estimated using a binomial generalized linear mixed model with threshold classification probability level of 0.7 and 0.5. Note that contingent composition for threshold probability level of 0.7 show proportion of samples that met the 0.7 threshold. Values in parentheses for the sample size (n) indicate the percentage of samples below the 0.7 threshold (unassigned). Data for year-classes 1998–2000 and 2011 are from Redding *et al*.^18^.

| **Category** | | **Contingent** | ***n*** | **Composition** | ***n*** | **Composition** |
| --- | --- | --- | --- | --- | --- | --- |
|  |  |  |  | **Prob. >0.7 (%)** |  | **Prob. >0.5 (%)** |
| **Year-class** | 1998 | Northern | 40 (4.8) | 60.0 | 42 | 59.5 |
|  |  | Southern |  | 40.0 |  | 40.5 |
|  | 1999 | Northern | 45 (15.1) | 46.7 | 53 | 50.9 |
|  |  | Southern |  | 53.3 |  | 49.1 |
|  | 2000 | Northern | 13 (13.3) | 76.9 | 15 | 80.0 |
|  |  | Southern |  | 23.1 |  | 20.0 |
|  | 2011 | Northern | 31(48.3) | 12.9 | 60 | 25.0 |
|  |  | Southern |  | 87.1 |  | 75.0 |
|  | 2012 | Northern | 24 (46.7) | 54.2 | 45 | 46.7 |
|  |  | Southern |  | 45.8 |  | 53.3 |
|  | 2013 | Northern | 32 (28.9) | 59.4 | 45 | 60.0 |
|  |  | Southern |  | 40.6 |  | 40.0 |
|  | 2014 | Northern | 23 (48.9) | 65.2 | 45 | 66.7 |
|  |  | Southern |  | 34.8 |  | 33.3 |
|  | 2015 | Northern | 28 (50.9) | 75.0 | 57 | 71.9 |
|  |  | Southern |  | 25.0 |  | 28.1 |
|  | 2016 | Northern | 6 (60.0) | 16.7 | 15 | 13.3 |
|  |  | Southern |  | 83.3 |  | 86.7 |
| **Age-class (Older)** | 2 | Northern | 47 (7.8) | 17.0 | 51 | 21.6 |
|  |  | Southern |  | 83.0 |  | 78.4 |
|  | 3 | Northern | 31 (8.8) | 90.3 | 34 | 88.2 |
|  |  | Southern |  | 9.7 |  | 11.8 |
|  | 4 | Northern | 18 (21.7) | 94.4 | 23 | 91.3 |
|  |  | Southern |  | 5.6 |  | 8.7 |
|  | 5 | Northern | 2 (0.0) | 100.0 | 2 | 100.0 |
|  |  | Southern |  | 0.0 |  | 0.0 |
| **Age-class (Recent)** | 2 | Northern | 41 (48.8) | 80.5 | 80 | 67.5 |
|  |  | Southern |  | 19.5 |  | 32.5 |
|  | 3 | Northern | 52 (45.3) | 48.1 | 95 | 48.4 |
|  |  | Southern |  | 51.9 |  | 51.6 |
|  | 4 | Northern | 51 (44.6) | 29.4 | 92 | 39.1 |
|  |  | Southern |  | 70.6 |  | 60.9 |
| **Size-class (cm)** | <29 | Northern | 63 (33.7) | 71.4 | 95 | 67.4 |
|  |  | Southern |  | 28.6 |  | 32.6 |
|  | 29–32 | Northern | 34 (64.2) | 41.2 | 95 | 46.3 |
|  |  | Southern |  | 58.8 |  | 53.7 |
|  | >32 | Northern | 47 (39.0) | 29.8 | 77 | 36.4 |
|  |  | Southern |  | 70.2 |  | 63.6 |
| **January** | MAB | Northern | – | – | – | – |
|  |  | Southern |  | – |  | – |
|  | SNE | Northern | 4 (20.0) | 0.0 | 5 | 20.0 |
|  |  | Southern |  | 100.0 |  | 80.0 |
|  | GB | Northern | 0 (100.0) | – | 2 | 0.0 |
|  |  | Southern |  | – |  | 100.0 |
|  | GOM | Northern | 4 (42.9) | 100.0 | 7 | 85.7 |
|  |  | Southern |  | 0.0 |  | 14.3 |
| **February** | MAB | Northern | 38 (39.7) | 52.6 | 63 | 60.3 |
|  |  | Southern |  | 47.4 |  | 39.7 |
|  | SNE | Northern | 11 (59.3) | 72.7 | 27 | 70.4 |
|  |  | Southern |  | 27.3 |  | 29.6 |
|  | GB | Northern | 12 (33.3) | 83.3 | 18 | 72.2 |
|  |  | Southern |  | 16.7 |  | 27.8 |
|  | GOM | Northern | – | – | – | – |
|  |  | Southern |  | – |  | – |
| **March** | MAB | Northern | 31 (42.6) | 64.5 | 54 | 53.7 |
|  |  | Southern |  | 35.5 |  | 46.3 |
|  | SNE | Northern | 3 (66.7) | 66.7 | 9 | 77.8 |
|  |  | Southern |  | 33.3 |  | 22.2 |
|  | GB | Northern | – | – | – | – |
|  |  | Southern |  | – |  | – |
|  | GOM | Northern | – | – | – | – |
|  |  | Southern |  | – |  | – |
| **April** | MAB | Northern | 6 (25.0) | 0.0 | 8 | 0.0 |
|  |  | Southern |  | 100.0 |  | 100.0 |
|  | SNE | Northern | 3 (72.7) | 66.7 | 11 | 45.5 |
|  |  | Southern |  | 33.3 |  | 54.5 |
|  | GB | Northern | 2 (66.7) | 50.0 | 6 | 33.3 |
|  |  | Southern |  | 50.0 |  | 66.7 |
|  | GOM | Northern | 10 (41.2) | 50.0 | 17 | 52.9 |
|  |  | Southern |  | 50.0 |  | 47.1 |
| **May** | MAB | Northern | – | – | – | – |
|  |  | Southern |  | – |  | – |
|  | SNE | Northern | – | – | – | – |
|  |  | Southern |  | – |  | – |
|  | GB | Northern | 6 (40.0) | 16.7 | 10 | 30.0 |
|  |  | Southern |  | 83.3 |  | 70.0 |
|  | GOM | Northern | 8 (33.3) | 0.0 | 12 | 16.7 |
|  |  | Southern |  | 100.0 |  | 83.3 |

**Figure caption**

**Supplementary Figure 1**

Time series (1970–2018) of Northwest Atlantic mackerel northern contingent (a) spawning stock biomass and (b) age-1 recruitment estimated by the censored-catch statistical catch-at-age model (DFO, 2019). Shaded regions indicate 95% confidence intervals. Data obtained from Fisheries and Oceans Canada (DFO).
